# Validation of Minimal Residual Disease as Surrogate Endpoint for Event-Free Survival in Childhood Acute Lymphoblastic Leukemia

**DOI:** 10.1093/jncics/pky069

**Published:** 2018-12-19

**Authors:** Stefania Galimberti, Meenakshi Devidas, Ausiliatrice Lucenti, Giovanni Cazzaniga, Anja Möricke, Claus R Bartram, Georg Mann, William Carroll, Naomi Winick, Michael Borowitz, Brent Wood, Giuseppe Basso, Valentino Conter, Martin Zimmermann, Stefan Suciu, Andrea Biondi, Martin Schrappe, Stephen P Hunger, Maria Grazia Valsecchi

**Affiliations:** 1 Center of Biostatistics for Clinical Epidemiology, School of Medicine and Surgery, University of Milano - Bicocca, Monza, Italy; 2 Department of Biostatistics, College of Medicine, Public Health and Health Professions, University of Florida, Gainesville, FL; 3 Fondazione Tettamanti, Monza, Italy; 4 Department of Pediatrics, University Medical Center Schleswig-Holstein, Campus Kiel, Kiel, Germany; 5 Institute of Human Genetics, University of Heidelberg, Heidelberg, Germany; 6 St. Anna Kinderspital, Wien, Austria; 7 Department of Pediatrics, New York University Langone Medical Center, New York, NY; 8 Department of Pediatrics, University of Texas Southwestern Medical Center, Dallas, TX; 9 Hematologic Pathology, Johns Hopkins University, Baltimore, MD; 10 Department of Laboratory Medicine, University of Washington, Seattle, WA; 11 Department SDB, University of Padua, Padova, Italy; 12 Pediatric Clinics, University of Milano - Bicocca, Monza, Italy; 13 Pediatric Hematology and Oncology, Hannover Medical School, Hannover, Germany; 14 EORTC, EORTC Headquarters, Brussels, Belgium; 15 Department of Pediatrics and the Center for Childhood Cancer Research, Children’s Hospital of Philadelphia and the Perelman School of Medicine at the University of Pennsylvania, Philadelphia, PA

## Abstract

**Background:**

The aim of this study was to assess whether minimal residual disease (MRD) at the end of induction front-line treatment can serve as a surrogate endpoint for event-free survival (EFS) in childhood B-lineage acute lymphoblastic leukemia.

**Methods:**

The analysis was based on individual data of 4830 patients from two large phase III trials that asked a randomized question on the effect of different corticosteroids (dexamethasone vs prednisone) during induction chemotherapy on EFS. The association between MRD classified in three ordered categories [negative = 0, low positive = (>0 and <5 × 10^−4^), and positive = (≥5 × 10^-4^)] and EFS at the individual and trial levels was evaluated with the meta-analytic approach based on the Plackett copula model. Centers within trial were grouped according to geographical area, and a total of 28 units were identified for the analysis.

**Results:**

MRD at the end of induction was a poor surrogate for treatment effect on EFS at the trial level, with $R_{\text{trial}}^{2}$ = 0.09 (95% confidence interval [CI] = 0.00 to 0.29), whereas at the individual level it was strongly associated with EFS, with an odds ratio of 3.90 (95% CI = 3.35 to 4.44) of failure for patients with higher compared with lower MRD levels. Additional sensitivity and relevant subgroup analyses confirmed these findings at both trial- and patient-level association.

**Conclusions:**

Although MRD is a robust biomarker highly predictive of outcome for individual patients, clinicians and regulatory bodies should be cautious in using early MRD response in the context of complex multiagent acute lymphoblastic leukemia therapy as an early surrogate endpoint to predict the effect of a randomized treatment intervention on long-term EFS.

Efficient clinical evaluation of new agents in oncology creates a need to consider novel clinical trial endpoints that facilitate assessment of efficacy of a new drug earlier than traditional endpoints such as event-free and overall survival (EFS and OS). This is particularly true for diseases with low event rates, such as pediatric acute lymphoblastic leukemia (ALL). To be a useful marker of efficacy, an early endpoint must be an accurate surrogate for the true endpoint of the clinical trial.

Minimal residual disease (MRD) is a powerful prognostic biomarker that measures the number of residual leukemic cells. The level of MRD remaining in bone marrow after completion of 1–3 months of multiagent chemotherapy is a well-known strong prognostic factor for EFS and OS in children with newly diagnosed ALL and is used routinely to assess treatment response and stratify treatment intensity and/or allocate patients to alternative therapies, such as hematopoietic stem cell transplant in first remission (1–7). However, it is not known whether early MRD response is a surrogate endpoint for EFS in evaluating efficacy of randomized treatment interventions. The consideration of MRD as surrogate trial endpoint is based on a high degree of biological plausibility and on robust evidence around its prognostic value, but no validation studies have been performed to date to assess the ability of MRD to capture the effect of randomized treatment interventions on EFS in pediatric ALL trials, as pointed out in a recent meta-analysis (3).

To our knowledge, this study addresses for the first time in childhood ALL the evaluation of surrogacy of MRD levels at the end of induction treatment. A meta-analytic approach was used on individual patient data from two large, randomized, phase III trials that compared efficacy of dexamethasone vs prednisone administered during induction chemotherapy.

## Methods

### Trials and Data

Two large, randomized, phase III studies comparing dexamethasone vs prednisone during induction therapy for children with B-precursor ALL were considered for surrogate marker evaluation. The two trials were conducted in Europe (AIEOP-BFM-ALL2000, n = 2955; NCT00613457 and NCT00430118) and North America, Australia, and New Zealand (COG-AALL0232, n = 1875; NCT00075725) (8,9). Data of children with measured MRD levels according to categories commonly used (5–7) were included in this individual meta-analysis and pooled according to a prespecified data structure. Patients in COG-AALL0232 were randomized for corticosteroids and either Capizzi or high-dose methotrexate in a 2 × 2 manner. For the purpose of this analysis, due to the significant quantitative interaction between methotrexate regimens and type of corticosteroid (8), we considered separately Capizzi (n = 945) and high-dose methotrexate (n = 930) groups as if they were two studies (COG-Capizzi and COG-High Dose MTX).

Patients were randomly assigned to receive either dexamethasone (10 mg/m^2^/d) or prednisone (60 mg/m^2^/d) in the induction phase of front-line treatment. Treatment duration was 3 weeks in AIEOP-BFM-ALL2000 (day 8–28) and 2 (dexamethasone; day 1–14) to 4 (prednisone; day 1–28) weeks in COG-AALL0232. The accrual was in 2000–2006 in AIEOP-BFM-ALL2000 and in 2004–2011 in COG-AALL0232; follow-up was updated to September 2011 and December 2014 for AIEOP-BFM-ALL2000 and COG-AALL0232, respectively, with an overall median follow-up of 8.1 years (9.1 years AIEOP-BFM and 6.9 years COG). Details of the design, study population, treatment protocol, and results of the randomized interventions have been previously reported (8,9).

### Statistical Methods

#### Surrogate Endpoint

The candidate surrogate endpoint was MRD at the end of the induction phase classified in three levels: negative (0), low positive (>0 and <5 × 10^-4^), and positive (≥5 × 10^-4^) according to the approach taken in AIEOP-BFM trials (5,6).

The trials included evaluation of MRD at induction day +33 (Ig/TCR/PCR-MRD in AIEOP-BFM-ALL2000) and +29 (flow-cytometry MRD in COG-Capizzi and COG-High Dose MTX), respectively, with a sensitivity of at least 10^-4^. Details on how MRD was assessed have been published previously (5–7).

#### True Endpoint

The primary clinical endpoint (ie, true endpoint) was EFS, defined as time from randomization to resistance at the end of induction, relapse at any site, second malignant neoplasm, death in remission, or last follow-up (censored observation), whichever occurred first. This definition of EFS differs from that previously reported in two aspects: 1) death during induction cannot be an event because this precludes MRD availability; 2) resistance is defined at day +29 in the COG trial and at day +33 in AIEOP-BFM, whereas in the AIEOP-BFM protocol the final evaluation of resistance was after consolidation therapy.

#### Evaluation of Surrogacy

The meta-analytic, multi-trial approach proposed by Burzykowski (10) was used to assess whether MRD is a valid surrogate endpoint for EFS. This approach relies on having a large number of trials, at least 10 when the trial level surrogacy is the quantity of interest (10). Here only two trials were available; yet because they were large multicenter trials, centers within each trial were grouped according to geographical area to define many trial units for the purpose of analysis. The approach grouped centers of neighboring regions in Italy and Germany and neighboring states/provinces in the United States/Canada to create trial units. The sites in Switzerland, Austria, Australia, and New Zealand constituted separate trial units. The procedure resulted in 28 trial units (6–9 trial units per group) with a median of 170 patients per trial unit (range 83–343 patients).

The association between MRD and EFS was investigated at both the patient and trial level (10) according to the intention-to-treat approach. Because the individual level surrogacy measures the association between the potential surrogate endpoint and the true endpoint (adjusting for treatment effect across all the trial units), it involves a multi-trial joint modelling of MRD and EFS. The bivariate Plackett copula model, with a parameter θ representing a global odds ratio, was adopted. The trial level surrogacy assesses the association between treatment effects on the surrogate and those on the true endpoint across all trial units and describes how well treatment effect on clinical endpoint can be predicted in a future trial based on the observed treatment effect on the surrogate. The quality of the surrogate at the trial level was assessed by using linear regression and by the coefficient of determination $R_{\text{trial}}^{2}$, adjusted for the presence of estimation error in treatment effects. An $R_{\text{trial}}^{2}$ value of at least 0.80 is required to declare trial level surrogacy (10). Because trials were split into trial units, the analysis was adjusted in the sense that, for each trial unit, separate treatment effects for the surrogate and true endpoints were considered, yet baseline hazards and shifts in the proportional odds models were assumed constant within trials. Thus, a proportional hazards model with Weibull trial-specific baseline hazard functions was used to estimate the effect of treatment on EFS, while treatment effect on MRD in three ordered categories was modelled by a proportional odds model with constant shifts within trials. No major departures from the parametric models assumptions were found. A SAS macro by Burzykowski (ressurCc) was used for the evaluation of surrogacy.

For descriptive purposes, trial/trial units treatment effects on MRD and EFS, along with the corresponding 95% confidence intervals (CI), were estimated through (cumulative) odds ratio (OR) and hazard ratio (HR) by the proportional odds model and the Cox model, respectively. A Cox model, stratified for trial, was used for estimating hazard ratios and testing treatment effects (score test) within each MRD category.

#### Sensitivity and Subpopulation Analyses

Additional sensitivity analyses were performed 1) censoring EFS at 2 and 3 years to evaluate short-term effects of MRD, 2) using two MRD categories (0 vs >0 and <5 × 10^-4^ vs >5 × 10^-4^) to simplify models and interpretation, and 3) considering relapse-free survival as additional true clinical endpoint. For this analysis, the adopted Cox model was on cause-specific hazard of relapse.

Surrogacy was further examined within subpopulations defined by key patient characteristics (eg, high-risk patients defined according to National Cancer Institute (NCI) criteria, defined as age ≥10 years or initial white blood cell count [WBC ≥50 × 10^9^/L] (11) and within children aged <10 years), by MRD protocol (PCR vs flow), and by study (excluding COG-Capizzi).

## Results

### Patient Description

A total of 4830 patients were considered, with a median age of 5.4 years and a median WBC count at diagnosis of 3.9 × 10^9^/L; 53.1% of patients were male. Induction treatments with prednisone or dexamethasone were equally distributed in the three studies; patient characteristics were balanced between the two treatment arms within each trial (Supplementary Table 1, available online). Patients from COG were older and had higher WBC levels, because this trial was limited to patients with NCI high-risk ALL.

### Outcomes

A summary of outcomes obtained for the surrogate and the true clinical endpoint are reported in Table 1, where the distribution of the three MRD categories and the 5-year EFS estimates are described by trial and treatment arm (Supplementary Table 2, available online shows the same figures in the 28 trial units). The MRD profiles showed a gradient: negative MRD was observed more frequently (range = 47.8%–67.5%) than low-positive (range = 15.7%–32.8%) and positive values (range = 16.1%–21.4%). Some heterogeneity in the three studies was present in treatment effect on MRD. The MRD levels were generally higher in the prednisone than in the dexamethasone group in AIEOP-BFM (OR = 0.82), and they were essentially the same in the two treatment groups in COG-HD MTX (OR = 0.99). In contrast, in COG-Capizzi, higher levels of MRD were observed in the dexamethasone group (OR = 1.39). In all trial arms, the 5-year EFS estimates were near 80%, ranging from 78.1% to 87.9%. The advantage of dexamethasone over prednisone was pronounced in AIEOP-BFM (HR = 0.72, 95% CI = 0.60 to 0.86) and COG-HD MTX (HR = 0.67, 95% CI = 0.49 to 0.90), whereas it was not statistically significant in COG-Capizzi (HR = 0.95, 95% CI = 0.73 to 1.24). Patients randomly assigned in the dexamethasone group generally had a lower unadjusted relapse rate, except for COG-Capizzi (crude relapse rate = 10.9% vs 16.0% in AIEOP-BFM, 10.9% vs 16.1% in COG-HD MTX, and 18.3% vs 16.4% in COG-Capizzi for dexamethasone and prednisone, respectively; Supplementary Table 3, available online).

**Table 1.** Observed treatment effects, with corresponding 95% CIs, on MRD and EFS in the main groups*

			EFS			MRD			
Group	Treatment	No.	No. EFS events	At 5 y (%)	HR (95% CI)†	Negative (%)	Low positive (%)	Positive (%)	OR (95% CI)‡
AIEOP-BFM	pred	1495	291	82.6		47.8	30.8	21.4	
	dexa	1460	210	87.9	0.72 (0.60 to 0.86)	51.1	32.8	16.1	0.82 (0.72 to 0.95)
COG-HD MTX	pred	472	104	79.7		67.0	16.7	16.3	
	dexa	458	69	86.2	0.67 (0.49 to 0.90)	67.5	15.7	16.8	0.99 (0.76 to 1.29)
COG-Capizzi	pred	475	110	78.1		66.5	16.2	17.3	
	dexa	470	106	78.9	0.95 (0.73 to 1.24)	57.9	21.7	20.4	1.39 (1.08 to 1.8)

* CI = confidence interval; dexa = dexamethasone; EFS = event-free survival; HR = hazard ratio; MRD = minimal residual disease; OR = odds ratio; pred = prednisone.

† HR of event for dexamethasone vs prednisone (reference category) and 95% CI estimated from a Cox model.

‡ (Cumulative) OR of high MRD for dexamethasone vs prednisone (reference category) and 95% CI estimated from a proportional odds model.

**Figure 1.**
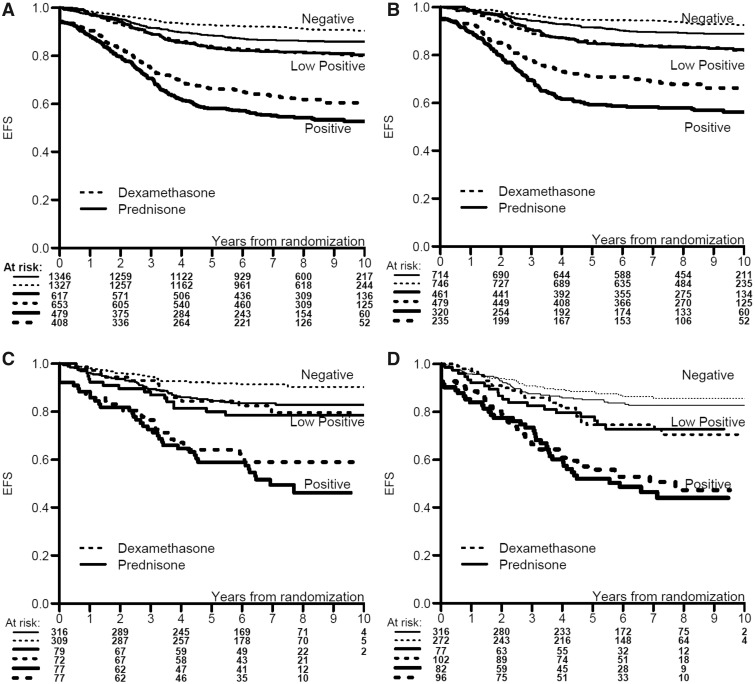
**A–D**) Event-free survival (EFS) curves by treatment within minimal residual disease (MRD) categories: **A**) overall, **B**) in AIEOP-BFM, **C**) in COG-HD, and **D**) in COG-Capizzi group.

Overall Kaplan-Meier EFS curves by treatment within MRD categories are shown in Figure 1A. A statistically significant difference between dexamethasone and prednisone in two out of the three MRD categories was detected (MRD negative: HR = 1.56, *P* = .0002; MRD low-positive: HR = 1.01, *P* = 0.92; MRD positive: HR = 1.30, *P* = .01). This indicates that MRD is not a surrogate for EFS according to Prentice’s definition (10) mainly because, within the two subgroups defined as negative and positive MRD levels, EFS curves by treatment are not superimposable. This pattern was consistently seen for the three studies (Figure 1, B–D).

### Analysis on Surrogacy

**Figure 2.**
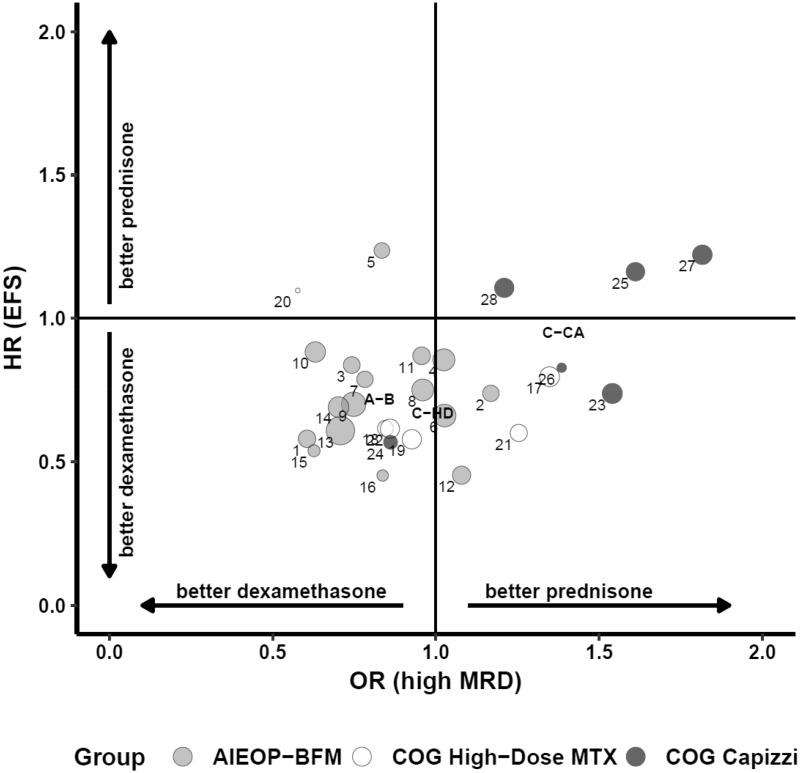
Trial level association between estimated (copula model) treatment effects (dexamethasone vs prednisone) on minimal residual disease (MRD) (odds ratio [OR]) vs event-free survival (EFS) (hazard ratio [HR]) in the 28 trial units that are represented with **circles** with size proportional to their sample size. **Shading** distinguishes trial units within main groups, whose overall effects are also represented.

The meta-analytic approach showed that MRD at the end of induction is a poor surrogate for treatment effect on EFS (Figure 2) at the trial level, with $R_{\text{trial}}^{2}$ = 0.09 (95% CI = 0.00 to 0.29), while at the individual level it showed a considerable prognostic association with EFS, after adjusting for treatment group, with an estimate of θ = 3.90 (95% CI = 3.35 to 4.44). This indicates that the odds of failure beyond a generic time *t* for larger MRD levels (low-positive and positive) were at least 3.9 times greater than the odds for lower MRD levels (negative).

Sensitivity analyses were conducted on short-term EFS (at 2 and 3 years), which might be more likely to be influenced by MRD than long-term outcome, with results highlighting a slightly higher prognostic association (θ = 4.30 and 3.98) and surrogacy at trial level of 0.02 and 0.05 (Table 2). Similar figures were obtained when two MRD categories were considered, contrasting negative vs any positivity or negative and low-positive vs highly positive measures. The analysis of MRD surrogacy on relapse-free survival resulted in a higher trial level association ($R_{\text{trial}}^{2}$ = 0.19) and a similar prognostic value of MRD (θ = 4.25) compared to remaining analyses.

**Table 2.** Results of sensitivity and subgroup analyses on surrogacy at patient and trial levels

		Patient-level association	Trial level association
Type of analysis	No.	θ_indiv_ (95% CI)	$R_{trial}^{2}$ (95% CI)
Overall	4830	3.90 (3.35 to 4.44)	0.09 (0.00 to 0.29)
Sensitivity analyses			
Censoring EFS at 2 years	4726*	4.30 (3.38 to 5.21)	0.02 (0.00 to 0.13)
Censoring EFS at 3 years	4830	3.98 (3.30 to 4.66)	0.05 (0.00 to 0.20)
MRD in 2 categories (0 vs >0)	4830	3.38 (2.85 to 3.91)	0.08 (0.00 to 0.28)
MRD in 2 categories (<5 × 10^4^ vs >5 × 10^4^)	4830	4.70 (3.95 to 5.46)	0.06 (0.00 to 0.23)
Relapse-free survival	4830	4.25 (3.63 to 4.86)	0.19 (0.00 to 0.45)
Subgroup analyses			
High-risk NCI criteria	2577	3.68 (3.01 to 4.34)	0.01 (0.00 to 0.06)
Age <10 y	3376	4.01 (3.30 to 4.72)	0.09 (0.00 to 0.29)
Excluding COG-Capizzi	3885	3.96 (3.34 to 4.59)	0.02 (0.00 to 0.12)
AIEOP-BFM only (ie, MRD-PCR only)	2955	4.10 (3.35 to 4.85)	0.01 (0.00 to 0.05)
COG only (ie, MRD-Flow only)	1875	3.77 (2.95 to 4.60)	0.13 (0.00 to 0.49)

* One trial unit (Switzerland) was excluded because there was no EFS event. CI = confidence interval; EFS = event-free survival; MRD = minimal residual disease; NCI = National Cancer Institute.

Additional analyses on relevant subgroups generally confirmed the previous findings both at the trial- and patient prognostic-level association. Results were similar in patients <10 years age and in high-risk patients defined according to NCI criteria (ie, excluding the non-high-risk AIEOP-BFM patients) both at the patient level (with values of θ 3.68–4.01; Table 2) and at the trial level association (with generally low values of $R_{\text{trial}}^{2}$; Table 2). Excluding the COG-Capizzi arm did not substantially change the results on surrogacy, and the same consideration holds when analysis was separately performed by MRD quantification methodology (Table 2).

## Discussion

To assess whether early MRD could serve as a surrogate for EFS in evaluating the effect of randomized treatment interventions in childhood B-precursor ALL, the currently available evidence from randomized trials on corticosteroids in induction was used. MRD was analyzed at the end of induction where it likely expresses more efficiently the effect of randomization. Individual data from the two largest collaborative trials conducted worldwide from 2000 to 2011 were pooled. Using a meta-analytic approach, we found that MRD, in three ordinal categories defined according to standard cut-points, is a poor surrogate for EFS at the trial level, indicating that the effect of the randomized corticosteroids (dexamethasone vs prednisone in induction) on the MRD level at end of induction does not reliably predict the effect of the intervention on EFS. In contrast, the analysis showed a strong association between end-induction MRD level and EFS time for individual patients, regardless of treatment, confirming the extremely strong prognostic effect of early MRD response on clinical outcome. These results were consistent across sensitivity and subgroup analyses.

This is the first attempt to our knowledge to explore individual and trial dimensions of MRD surrogacy in the context of childhood ALL. Two studies recently performed in chronic lymphocytic leukemia (12) and in multiple myeloma (13) corroborated the prognostic impact of MRD but omitted the evaluation of trial level association, which is mandatory for supporting the use of MRD as a substitute endpoint for long-term clinical outcomes, as pointed out in two commentaries from the NCI Biometric Research Branch (14,15). The strong prognostic value of achieving MRD negativity at the end of induction therapy was demonstrated in both pediatric and adult patients with ALL in a recent meta-analysis (3) and strongly confirmed in the current study.

The key question asked in the current analyses is whether end-induction MRD could serve as a potential early endpoint that might be used for accelerated approval of a new drug, which requires that the effect of the randomized intervention on MRD accurately reflects the effect of that intervention on EFS to confirm benefit on EFS or OS (16). The statistical meta-analytic approach to validation we used is recognized as a standard method to address this issue. The included trials are different in some aspects, for example, eligibility criteria, induction regimens, and post induction therapies. The presence of these diversities, which implies additional sources of heterogeneity in the analysis, is a strength in terms of generalizability of results of the validation process to future clinical trials. More importantly, the methods used for MRD measurement are different (PCR in Europe and FCM in North America) but quite concordant (17,18).

We performed the analysis considering MRD as an ordinal surrogate endpoint, defining the three MRD classes commonly used in clinical practice (ie, MRD negative, low-positive, positive), because categorical data were available only for BFM (5). No further investigation was carried out on the subset of MRD data as a continuous variable, because this requires the development of an appropriate statistical method able to validate a highly skewed marker with a high proportion of zero values as a surrogate.

The evaluation of surrogacy with a multi-trial approach ideally requires a relatively high number of trials. We acknowledge here the limitation that with two trials, we may not have captured all the relevant variability needed to optimally evaluate the ability of the effect on a putative surrogate to predict the effect on a definitive endpoint (in a new trial in which only the surrogate endpoint would be measured). However, given the large number of patients and centers in the trials, we were able to artificially increase the trial level sample size by dividing the trials into many trial subunits in which treating centers were aggregated according to extended geographical areas sharing the same reference population (10,19). Other methods for defining trial units, for example, small vs large centers, would likely introduce bias and only a random-based grouping could be an alternative. This, however, would lose the geographical reference population. The hierarchical structure of our data (geographical areas within trial) was accounted for in the analysis by adjusting the models to account for the common trial. As shown by Renfro et al. (20), the analysis based on trial units nested within trials can potentially inflate the estimate of trial-level surrogacy, suggesting the use of the surrogate when it is not correct, which is not the case in our setting. Also, the variability in treatment effects, which surely increased the background noise, is relevant for the validation process because a broad range of treatment effects on both the surrogate and the true endpoint is required.

We acknowledge two additional limitations that might have contributed to dilute the potential of MRD in being a valid surrogate in the context of front-line ALL treatments. The effect of type of corticosteroid on MRD distribution at the end of induction is relatively limited in size and not uniformly in favor of dexamethasone in the three groups. On the other hand, treatment effect on the true endpoint is coherent across groups although limited in size in one group (Table 1). Conditions for surrogacy are, however, that treatments must have a clear effect both on surrogate and true endpoint, at least in some trials, and that the effects must be concordant. Moreover, post-induction treatment complexity and intensity, partly tailored on MRD itself as a key criterion to modulate the intensity of subsequent therapy, may have also confounded the effect of induction treatments on EFS. This raises concern about the likely success of pursuing the utility of MRD as a surrogate endpoint rather than focusing on its current use as a powerful prognostic factor. It is also important to acknowledge that the above limitations did not impact the powerful value of MRD as a prognostic biomarker at the individual patient level.

What lessons can be learned from this study? First, a marker that is a strong prognostic factor cannot be automatically assumed to be a surrogate, which goes against a common belief that is hard to overcome in the medical community (12,13). Indeed, surrogacy needs to be specifically evaluated also at the trial level (14,15): if a potential surrogate endpoint such as MRD is highly correlated with a true endpoint, then a beneficial (or detrimental) effect on the marker between randomized groups would imply a beneficial (or detrimental) effect in EFS between these groups. If this is not the case, as in our context, the marker remains prognostic in both treatments, but a relevant difference between treatments cannot be assumed on the true endpoint. Second, the fact that a marker is not a surrogate for given drugs or a class of drugs in a given disease should not prevent the investigation of the same marker for other drugs or in other diseases, owing to possible different drug mechanisms of action within different treatment schedules. In particular, the use of MRD for screening new drugs in terms of activity may still be explored, especially when randomized, early-phase studies adopt an add-on design (a standard chemotherapy regimen with or without addition of a new drug). Third, in a complex and long treatment protocol, such as the one adopted in ALL, the timing of the marker measurement might be important. For instance, there could still be hope for surrogacy of an MRD measurement made later in the treatment course (although this might require standardization of the therapeutic approach before the MRD measurement). Fourth, it is difficult to establish surrogacy of a marker measured early in time on a long-term outcome, in particular when therapeutic decisions are taken based on this marker. A bias in either direction might affect surrogacy, with no statistical methods available to adjust for it. For instance, if more aggressive therapy to MRD-positive patients provides a benefit, treatment effect on EFS, along with surrogacy association, would be attenuated and the opposite would happen if treatment is detrimental. Finally, the need for many large, randomized trials for evaluating surrogacy may limit the application in rare diseases. In the context of pediatric ALL, this limitation may pave the way to future validation studies on MRD, which expand the collection of trials to randomized questions on different classes of drugs. This broader approach may be informative unless increased variability further diminishes the surrogacy association.

In conclusion, despite the strong patient-level impact of end-induction MRD as a prognostic biomarker, the results of our study suggest that clinicians and regulatory bodies should be cautious in using early MRD response in the context of complex multiagent ALL therapy as an early surrogate endpoint to predict the effect of a randomized treatment intervention on long-term EFS.

## Funding

This work was partially supported by the Associazione Italiana Ricerca sul Cancro AIRC Investigator Grant (grant no. 2013-14634 to MGV).

## Notes

Affiliations of authors: Center of Biostatistics for Clinical Epidemiology, School of Medicine and Surgery, University of Milano - Bicocca, Monza, Italy (SG, AL, MGV); Department of Biostatistics, College of Medicine, Public Health and Health Professions, University of Florida, Gainesville, FL (MD); Fondazione Tettamanti, Monza, Italy (GC); Department of Pediatrics, University Medical Center Schleswig-Holstein, Campus Kiel, Kiel, Germany (AM, MS); Institute of Human Genetics, University of Heidelberg, Heidelberg, Germany (CRB); St. Anna Kinderspital, Wien, Austria (GM); Department of Pediatrics, New York University Langone Medical Center, New York, NY (WC); Department of Pediatrics, University of Texas Southwestern Medical Center, Dallas, TX (NW); Hematologic Pathology, Johns Hopkins University, Baltimore, MD (MB); Department of Laboratory Medicine, University of Washington, Seattle, WA (BW); Department SDB, University of Padua, Padova, Italy (GB); Pediatric Clinics, University of Milano - Bicocca, Monza, Italy (VC, AB); Pediatric Hematology and Oncology, Hannover Medical School, Hannover, Germany (MZ); EORTC, EORTC Headquarters, Brussels, Belgium (SS); Department of Pediatrics and the Center for Childhood Cancer Research, Children’s Hospital of Philadelphia and the Perelman School of Medicine at the University of Pennsylvania, Philadelphia, PA (SPH).

We acknowledge Daniela Silvestri for her valuable contribution in preparing the AIEOP database. We also acknowledge the contribution of the referees who greatly helped to improve the clarity of the article.

Clinical trial numbers: NCT00613457, NCT00430118, and NCT00075725.

## Supplementary Material

Supplementary Data
