# Supplementary material for: Validation of Minimal Residual Disease as Surrogate Endpoint for Event-Free Survival in Childhood Acute Lymphoblastic Leukemia
Source: JNCI Cancer Spectr. 2018 Dec 19;2(4):pky069. doi: 10.1093/jncics/pky069 (PMC6649800; doi:10.1093/jncics/pky069)
Supplement: Supplementary Data [file pky069_supp.pdf]

Supplementary Table 1: Patients characteristics at baseline by treatment in the main trials.

	AIEOP-BFM ALL2000 (N=2955)				COG AALL0232* (N=1875)					
	**dexa**		**pred**		**dexa**		**pred**		**Total**	
**Characteristic**	**No.**	**(%)**	**No.**	**(%)**	**No.**	**(%)**	**No.**	**(%)**	**No.**	**(%)**
**Total**	1460	49·4	1495	50·6	928	49·5	947	50·5	4830	(100)
**Sex**										
Male	804	55·1	757	50·6	496	53·4	506	53·4	2563	53·0
Female	656	44·9	738	49·4	432	46·6	441	46·6	2267	47·0
**Age (years)**										
1-9	1256	86·0	1277	85·0	420	45·3	423	44·7	3376	69·9
≥10[#]	204	14·0	218	15·0	508	54·7	524	55·3	1454	30·1
Median (min-max)	4·5	(1·0-17·8)	4·5	(1·0-17·9)	10·7	(1·0-25·7)	10·9	(1·0-30·07)	5·4	(1-30)
**WBC count (x10^9/L)**										
< 50	1252	85·8	1283	85·8	429	46·2	444	46·9	3408	70·6
≥ 50	208	14·2	212	14·2	499	53·8	503	53·1	1422	29·4
Median (min-max)	9·4	(0·4-567)	9·8	(0·1-875)	54·0	(0·3-1000)	53·3	(0·3-1306)	13·9	(0·1-1306)
**CNS status**										
Yes	23	1·6	26	1·7	0	0	0	0	49	1·0
No	1416	97·0	1436	96·1	928	100	946	99·9	4726	97·9
NK	21	1·4	33	2·2	0	0	1	0·1	55	1·1
**ETV6-RUNX1**										
Yes	382	26·2	372	24·9	146	15·7	149	15·7	1049	21·7
No	962	65·9	1022	68·4	628	67·7	633	66·8	3245	67·2
NK	116	7·9	101	6·8	154	16·6	165	17·4	536	11·1
**MLL/ENL or t(4;11)^**										
Yes	4	0·3	13	0·9	22	2·4	23	2·4	62	1·3
No	1367	93·6	1409	94·2	712	76·7	711	75·1	4199	86·9
NK	89	6·1	73	4·9	194	20·9	213	22·5	569	11·8

Abbreviations: dexa=dexamethasone; pred=prednisone; SD=standard deviation.

[*] The distributions were similar between COG-Capizzi and COG-High Dose MTX arm.

[#] Age eligibility criteria for AIEOP-BFM and COG were 1-17 and 1-21 years at diagnosis, respectively

^ In COG where MLL FISH was performed it refers to KMT2A.

Supplementary Table 2: Observed treatment effects, with corresponding 95% CIs, on EFS and MRD in the 28 trial-units defined according to geographical area.

				EFS			MRD			
**Group**	**Trial-Unit**	**Treatment**	**No.**	**No. EFS events**	**5 yrs (%)**	**HR (95% CI)^**	**Negative (%)**	**Low Positive (%)**	**Positive (%)**	**OR (95% CI)***
AIEOP	1	pred	77	16	80·4		41·6	35·1	23·4	
		dexa	81	10	91·4	0·55 (0·25 to 1·21)	58·0	32·1	9·9	0·47 (0·26 to 0·86)
	2	pred	86	19	79·7		47·7	24·4	27·9	
		dexa	60	9	88·2	0·62 (0·28 to 1·37)	38·3	43·3	18·3	1·06 (0·58 to 1·94)
	3	pred	79	15	83·5		41·8	35·4	22·8	
		dexa	71	12	87·3	0·88 (0·41 to 1·88)	52·1	38·0	9·9	0·57 (0·31 to 1·06)
	4	pred	103	26	77·4		40·8	33·0	26·2	
		dexa	119	19	85·6	0·61 (0·34 to 1·10)	44·5	37·0	18·5	0·77 (0·47 to 1·27)
	5	pred	70	14	82·9		45·7	40·0	14·3	
		dexa	69	17	81·2	1·25 (0·62 to 2·53)	53·6	24·6	21·7	0·91 (0·48 to 1·7)
	6	pred	124	34	75·7		49·2	26·6	24·2	
		dexa	107	14	87·8	0·44 (0·24 to 0·83)	44·9	33·6	21·5	1·07 (0·66 to 1·73)
	7	pred	74	13	83·8		43·2	40·5	16·2	
		dexa	72	12	87·5	0·92 (0·42 to 2·02)	52·8	31·9	15·3	0·74 (0·4 to 1·36)
BFM	8	pred	112	15	86·6		34·8	45·5	19·6	
		dexa	107	16	88·6	1·14 (0·56 to 2·3)	44·9	41·1	14·0	0·66 (0·4 to 1·09)
	9	pred	127	24	81·8		52·8	24·4	22·8	
		dexa	135	19	86·5	0·73 (0·40 to 1·33)	54·1	28·9	17·0	0·87 (0·55 to 1·39)
	10	pred	87	18	80·7		55·2	20·7	24·1	
		dexa	110	18	87·1	0·76 (0·40 to 1·47)	60·0	25·5	14·6	0·74 (0·43 to 1·28)
	11	pred	79	15	84·8		48·1	27·9	24·1	
		dexa	82	13	86·3	0·86 (0·41 to 1·80)	47·6	35·4	17·1	0·89 (0·5 to 1·6)
	12	pred	85	17	85·6		45·9	31·8	22·4	
		dexa	83	8	90·3	0·45 (0·20 to 1·05)	47·0	26·5	26·5	1·05 (0·6 to 1·85)
	13	pred	185	31	84·1		53·0	31·4	15·7	
		dexa	158	19	88·5	0·70 (0·40 to 1·24)	55·1	31·0	13·9	0·91 (0·6 to 1·37)
	14	pred	94	16	87·1		53·2	24·5	22·3	
		dexa	107	14	89·6	0·80 (0·39 to 1·64)	53·3	36·5	10·3	0·81 (0·47 to 1·38)
	15	pred	61	11	83·6		52·5	26·2	21·3	
		dexa	47	5	91·3	0·54 (0·19 to 1·56)	57·5	31·9	10·6	0·72 (0·34 to 1·49)
	16	pred	52	7	88·1		57·7	26·9	15·4	
		dexa	52	5	92·2	0·65 (0·20 to 2·03)	51·9	28·9	19·2	1·27 (0·61 to 2·67)
COG-High Dose MTX	17	pred	110	19	83·0		72·7	16·4	10·9	
		dexa	80	14	84·4	1·03 (0·52 to 2·05)	60·0	20·0	20·0	1·82 (1 to 3·33)
	18	pred	63	9	88·1		65·1	14·3	20·6	
		dexa	77	11	86·6	0·96 (0·40 to 2·33)	71·4	13·0	15·6	0·74 (0·37 to 1·49)
	19	pred	88	23	75·4		64·8	12·5	22·7	
		dexa	94	12	87·9	0·49 (0·24 to 0·98)	69·2	13·8	17·0	0·79 (0·43 to 1·45)
	20	pred	43	12	76·2		62·8	18·6	18·6	
		dexa	40	9	79·6	0·86 (0·36 to 2·04)	77·5	10·0	12·5	0·51 (0·2 to 1·32)
	21	pred	76	19	79·6		69·7	17·1	13·2	
		dexa	79	11	87·9	0·52 (0·25 to 1·09)	62·0	19·0	19·0	1·44 (0·75 to 2·76)
	22	pred	92	22	76·0		63·0	21·7	15·2	
		dexa	88	12	87·1	0·53 (0·26 to 1·07)	69·3	15·9	14·8	0·79 (0·43 to 1·45)
COG Capizzi	23	pred	89	17	85·9		66·3	19·1	14·6	
		dexa	103	19	82·7	0·98 (0·51 to 1·88)	56·3	22·3	21·4	1·54 (0·87 to 2·72)
	24	pred	64	12	81·1		62·5	12·5	25·0	
		dexa	58	8	87·4	0·72 (0·29 to 1·75)	70·7	12·1	17·2	0·67 (0·32 to 1·42)
	25	pred	80	20	73·5		77·5	12·5	10·0	
		dexa	91	24	76·2	1·05 (0·58 to 1·91)	55·0	24·2	20·9	2·75 (1·42 to 5·29)
	26	pred	47	14	68·7		55·3	19·2	25·5	
		dexa	46	9	80·9	0·60 (0·26 to 1·40)	56·5	28·3	15·2	0·81 (0·37 to 1·79)
	27	pred	98	24	79·2		67·4	19·4	13·3	
		dexa	87	24	74·5	1·13 (0·64 to 1·98)	51·7	24·1	24·1	1·96 (1·1 to 3·49)
	28	pred	97	23	76·2		65·0	14·4	20·6	
		dexa	85	22	74·7	1·04 (0·58 to 1·86)	61·2	18·8	20·0	1·12 (0·62 to 2·02)

Abbreviations: dexa=dexamethasone; pred=prednisone; MRD=Minimal Residual Disease; EFS=Event Free Survival; OR=Odds Ratio; Hazard Ratio=HR; CI=Confidence Interval

*(cumulative) Odds Ratio of high MRD for dexamethasone vs prednisone (reference category) and 95% CI estimated from a proportional odds model

^Hazard Ratio of event for dexamethasone vs prednisone (reference category) and 95% CI estimated from a Cox model

Supplementary Table 3: Distribution of the EFS events by treatment in the 3 groups.

Group	dexa		pred		Total	
**Event type**	**No.**	**%**	**No.**	**%**	**No.**	**%**
**AIEOP-BFM**	1460		1495		2955	
**Total n. of events**	210	14·4	291	19·5	501	17·0
**Resistance***	13	0·9	16	1·1	29	1·0
**Relapse**	159	10·9	239	16·0	398	13·5
BM±other	*134*	*9·2*	*196*	*13·1*	*330*	*11·2*
CNS isolate	*14*	*1·0*	*19*	*1·3*	*33*	*1·1*
Testis isolate	*6*	*0·4*	*21*	*1·4*	*27*	*0·9*
Other	*5*	*0·3*	*3*	*0·2*	*8*	*0·3*
**SMN**	21	1·4	16	1·1	37	1·3
**Death**	17	1·2	20	1·3	37	1·3
**Alive in CR**	1250	85·6	1204	80·5	2454	83
**COG-High Dose MTX**	458		472		930	
**Total n. of events**	69	15·1	104	22·0	173	18·6
**Resistance***	6	1·3	6	1·3	12	1·3
**Relapse**	50	10·9	76	16·1	126	13·5
BM±other	*39*	*8·5*	*54*	*11·4*	*93*	*10·0*
CNS isolate	*8*	*1·7*	*17*	*3·6*	*25*	*2·7*
Testis isolate	*2*	*0·4*	*1*	*0·2*	*3*	*0·3*
Other	*1*	*0·2*	*4*	*0·8*	*5*	*0·5*
**SMN**	3	0·7	8	1·7	11	1·2
**Death**	10	2·2	14	3·0	24	2·6
**Alive in CR**	389	84·9	368	78·0	757	81·4
**COG-Capizzi**	470		475		945	
**Total n. of events**	106	22·5	110	23·2	216	22·9
**Resistance***	7	1·5	8	1·7	15	1·6
**Relapse**	86	18·3	78	16·4	164	17·4
BM±other	*63*	*13·4*	*50*	*10·5*	*113*	*12·0*
CNS isolate	*17*	*3·6*	*20*	*4·2*	*37*	*3·9*
Testis isolate	*3*	*0·6*	*2*	*0·4*	*5*	*0·5*
Other	*3*	*0·6*	*6*	*1·3*	*9*	*1·0*
**SMN**	2	0·4	7	1·5	9	1·0
**Death**	11	2·3	17	3·6	28	3·0
**Alive in CR**	364	77·5	365	76·8	729	77·0

Abbreviations: dexa=dexamethasone; pred=prednisone; BM=bone marrow; CNS=central nervous system; SMN=secondary malignant neoplasm; CR= complete remission

* Defined as no CR at the end of induction (day +33 in AIEOP-BFM and day +29 in COG trial)
